# Altered Brain Topological Property Associated With Anxiety in Experimental Orthodontic Pain

**DOI:** 10.3389/fnins.2022.907216

**Published:** 2022-05-11

**Authors:** Feifei Zhang, Fei Li, Hong Yang, Yu Jin, Wenli Lai, Graham J. Kemp, Zhiyun Jia, Qiyong Gong

**Affiliations:** ^1^Department of Radiology, Huaxi MR Research Center (HMRRC), West China Hospital of Sichuan University, Chengdu, China; ^2^Department of Radiology, First Hospital of Shanxi Medical University, Taiyuan, China; ^3^Research Unit of Psychoradiology, Chinese Academy of Medical Sciences, Chengdu, China; ^4^State Key Laboratory of Oral Disease, Department of Orthodontics, West China School of Stomatology, Sichuan University, Chengdu, China; ^5^Liverpool Magnetic Resonance Imaging Centre (LiMRIC) and Institute of Life Course and Medical Sciences, University of Liverpool, Liverpool, United Kingdom; ^6^Department of Nuclear Medicine, West China Hospital of Sichuan University, Chengdu, China; ^7^Functional and Molecular Imaging Key Laboratory of Sichuan University, Chengdu, China

**Keywords:** orthodontic pain, rs-fMRI, graph theory, functional connectivity, anxiety

## Abstract

**Background:**

Orthodontic pain is orofacial pain caused by tooth movement. Anxiety is a strong predictor of the severity of such pain, but little is known about the underlying neuropsychological mechanisms of such effects. The purpose of this study was to investigate the effect of orthodontic pain on brain functional networks and to define the mediating role of anxiety in orthodontic pain and brain function.

**Methods:**

Graph theory-based network analyses were applied to brain functional magnetic resonance imaging data from 48 healthy participants exposed to 24 h orthodontic pain stimuli and 49 healthy controls without any stimulation.

**Results:**

In the experimental orthodontic pain stimulation, brain functional networks retained a small-world organization. At the regional level, the nodal centrality of ipsilateral brain nodes to the pain stimulus was enhanced; in contrast the nodal centrality of contralateral brain areas was decreased, especially the right mid-cingulate cortex, which is involved in pain intensity coding. Furthermore, anxiety mediated the relationship between nodal efficiency of mid-cingulate cortex and pain severity.

**Conclusion:**

The results illuminate the neural mechanisms of orthodontic pain by revealing unbalanced hemispherical brain function related to the unilateral pain stimulation, and reveal clinically exploitable evidence that anxiety mediates the relationship between nodal function of right mid-cingulate cortex and orthodontic pain.

## Introduction

Orofacial pain, a kind of chronic pain (Long et al., 2016) caused by tooth movement, is a common side effect of orthodontic treatment. The main neural pathway is through the trigeminal nerve to the thalamus and then to the cerebral sensory cortex (Jantsch et al., 2005; Long et al., 2016). Nearly 95% of patients suffer from some degree of discomfort to pain during orthodontic procedures (Erdinc and Dincer, 2004), and this is the commonest reason that patients want to discontinue orthodontic treatment (Monk et al., 2017). Understanding the underlying brain neural mechanisms is important to help to alleviate pain and improve the results of treatment.

Pain is associated with the co-activation of numerous brain regions. In a similar model of experimental orthodontic pain (Yang et al., 2015), we recently reported altered functional connectivity (FC) in the thalamus and cingulum (Zhang et al., 2020). In other kinds of orofacial pain, the insula, mid-cingulate cortex (MCC) and frontal gyri are frequently reported as having increased functional activity even under weak pain stimulation (Jantsch et al., 2005; Youssef et al., 2014; Yin et al., 2020).

The graph-theoretical approach to analyzing large-scale networks is a powerful tool to explore brain organization (Rubinov and Sporns, 2010). The topological organization of the network is important in brain function: both global and nodal topological properties influence the efficiency of information transmission between brain regions (Rubinov and Sporns, 2010). In the presence of pain, the internal network topology of the brain is partially reconstructed (Farmer et al., 2012). Studies of patients with chronic pain have shown that the normal ‘small-world’ brain network organization is present, with no significant difference in global properties compared with healthy controls (HCs) (Kaplan et al., 2019; De Pauw et al., 2020). However, brain nodal properties were significantly different from HCs in the middle prefrontal cortex, anterior cingulate cortex, somatosensory cortex, temporal gyrus and insula (Kaplan et al., 2019; Tu et al., 2019). Beyond the classical neural model of orthodontic pain, there is evidence that several brain regions and networks with an essential role in emotion and cognition are involved in the pain process (Jahn et al., 2016; Reicherts et al., 2017; Wang et al., 2017). For example, altered function of MCC, insula, and regions in default mode network is related to pain, but also to emotional and cognitive function changes (Jahn et al., 2016; Reicherts et al., 2017; Wang et al., 2017). This suggests a reason to explore the workings of large-scale brain networks in orthodontic pain, both to define the pathophysiology and to explore the potential for treatment and prevention. However, it is still unclear how orthodontic pain influences brain functional topology. The first aim of this study was therefore to use an experimental orthodontic pain model to explore pain-related changes in gray matter functional networks based on rs-fMRI data and graph theory analysis.

Orthodontic pain not only hinders the orthodontic treatment (Erdinc and Dincer, 2004; Wang et al., 2015; Monk et al., 2017) but has harmful effects on physical and mental health (Sari et al., 2005). It is therefore important to determine reliable psychosocial predictors of orthodontic pain, in order to formulate prevention strategies and interventions to reduce pain. There is extensive evidence that dental anxiety is an emotional feature of patients with facial pain and also one of the major barriers to dental care (Michelotti et al., 2012; Fillingim et al., 2013; Chow and Cioffi, 2019). Anxiety is related to the frequency of orthodontics, and both are affected by orthodontic pain (Chow and Cioffi, 2019). Further research shows that patients’ anxiety during orthodontic treatment may also be an effective predictor of pain (Sari et al., 2005; Fillingim et al., 2013). Based on the previous research evidence of anxiety in orthodontics and pain, we believe that studying the relationship between anxiety and pain in subjects with orthodontic treatment may be important to alleviate orthodontic pain from the perspective of behavioral psychology. In addition, neuroimaging studies reveal that the anxiety-magnified pain intensity is dependent on neural activation in the thalamus, insula, and MCC (Bushnell et al., 2013; Reicherts et al., 2017), which is also reported in orthodontic pain. Given these findings, the second aim of this study was to elucidate, by means of a mediation analysis, the neuropsychological mechanisms of how anxiety affects orthodontic pain through gray matter function.

In light of the literature findings outlined above, we hypothesized (1) that small-world organization of the brain functional networks would be preserved in orthodontic pain participants, but (2) that nodal topological properties would show differences from controls in the insula, cingulate cortex and somatosensory cortex; also (3) the level of anxiety would be positively related to orthodontic pain, and further (4) it would play a mediating role in the relationship between brain function and orthodontic pain.

## Materials and Methods

### Participants

Fifty-two subjects were enrolled in the experimental orthodontic pain group, and 49 subjects in the control group. Inclusion criteria were: age 18-60 years, Chinese Han nationality, no history of orthodontics. Exclusion criteria were: (a) medication for pain; (b) left-handedness; (c) any history of major illness, such as somatic or psychiatric disorders, neurological disorders, or severe head trauma with loss of consciousness; (d) claustrophobia or any contraindications for magnetic resonance imaging (MRI) scans, including pregnancy; (e) history of current serious medical problems. **Forty-two out of the 52 subjects in the pain group and all 49 controls were reported in our previous study (Zhang et al., 2020).**

### Experimental Protocol

Patients usually experience pain about 12 h after application of orthodontic force, the pain peaking after about 24 h (Johal et al., 2018). In this study, experimental orthodontic pain was introduced in healthy subjects by an elastic separator applied, with the aid of dental floss, between the right first and second molar on the mesial and distal side for 24 h. The separator is stretched around the contact area of the teeth, pushing them apart and thus giving rise to orthodontic pain. After 24 h, MRI scan was performed with the elastic separator *in situ*, and the elastic separator was removed immediately afterward.

Participants completed the visual analog pain scale (VAS) (Myles et al., 2017) and the state anxiety inventory (STAI) (Ramanaiah et al., 1983) before the application of the elastic separator and again after 24 h, immediately before MRI scanning. VAS pain scores range from 0 to 100: the higher the score, the more severe the pain (Myles et al., 2017). The STAI is a 4-point Likert-type 20-item self-report questionnaire with options ranging from 1 (never) to 4 (always): a higher overall score indicates more severe anxiety. In the control group, no elastic separator was inserted and no measurements of VAS and STAI were obtained. **Before MRI scan, we orally confirmed that there was no pain or discomfort in the control group.**

### Brain MRI Data Acquisition

The rs-fMRI data were acquired using a Siemens 3.0 Tesla MR system (Tim Trio; Siemens Healthineers, Erlangen, Germany). Foam blocks were used to minimize head movements. MRI scanning was performed with the elastic separator *in situ* for the subjects with orthodontic pain, and removed immediately after the scanning. The rs-fMRI images, scanning parallel to the anterior and posterior commissures, were acquired with an echo-planar sequence: echo time: 30 ms; repetition time: 2,000 ms; flip angle: 90°; slice thickness: 5 mm, 30 slices; matrix: 64 × 64 mm^2^; field of view (FOV): 240 mm × 240 mm; voxel size: 3.75 mm × 3.75 mm × 5 mm. During the 410 s rs-fMRI acquisition, participants were instructed to close their eyes and to try not to think about anything. High-resolution T1-weighted three-dimensional images were acquired using a magnetization-prepared rapid gradient-echo sequence: echo time: 2.26 ms; repetition time: 1900 ms; flip angle: 9°; 175 axial slices with slice thickness: 1 mm; matrix: 256 mm × 256 mm; FOV: 240 mm × 240 mm; single-voxel size: 1 mm × 1 mm × 1 mm. The T1 weighted images were reviewed by a radiologist for visible abnormalities.

### Image Preprocessing

Structural and functional MRI data were preprocessed using SPM8^1^ and DPARSF^2^ in MATLAB 2013b, in the following steps: removal of the first 10 time-points; slice-timing correction; realignment (head motion was required to be < 2.5 mm translation and < 2.5° rotation); regression out of nuisance signals; 24-parameter motion correction; removal of white matter and cerebrospinal fluid signals; reduction of effects of head movement by scrubbing the motion: ‘spikes’ with a high framewise displacement > 1 mm (Power et al., 2012) were regressed out as a separate regressor; segmentation of high-resolution brain structural images into gray matter, white matter and cerebrospinal fluid; registration of the functional images to each individual’s 3D T1 structural images; normalization of functional images into Montreal Neurological Institute space with voxel size of 3 mm × 3 mm × 3 mm; smoothing using a 4 mm full-width at half-maximum Gaussian kernel; outline detection; bandpass filter with a frequency window of 0.01 to 0.10 Hz.

### Brain Network Construction and Topological Properties Calculation

The 246-template (Fan et al., 2016) was used to constructing each individual brain functional network in GRETNA^3^ by calculating the Pearson correlations of the time series of the rs-fMRI data signal among the 246 brain regions. We first binarized the FC matrices with a generated sparsity thresholds ranging from 0.24 to 0.35 with a step size of 0.01, determined based on previous standards and normalized networks to have the same number of edges (Zhang et al., 2011). We calculated the global and nodal topologic properties at each sparsity, and then the area under the curve (AUC) across the sparsity range to provide an overall value for the topological metrics. The global properties include small-world parameters (Watts and Strogatz, 1998): the clustering coefficient, characteristic path length, normalized clustering coefficient, normalized characteristic path length, and small-worldness (for normalization, 100 random graphs with the same number of nodes and edges were constructed as the baseline for each network). The property of small-worldness represents the optimum balance between information processing separation and integration in human brain networks (Watts and Strogatz, 1998). The other global properties are network efficiency parameters (Latora and Marchiori, 2001): local efficiency and global efficiency. The nodal properties were nodal degree, nodal efficiency, and nodal betweenness. These reflect the importance of the node to the global network functioning (Rubinov and Sporns, 2010).

### Statistical Analysis

Independent-sample *t*-tests were performed to compare demographic data (apart from the sex ratio, analyzed by a chi-square test), clinical data, and topological properties between the orthodontic pain and control groups. In the pain group, partial correlation analyses were performed between the questionnaire scores and AUC of the topological properties that exhibited significant between-group differences, using age and sex as covariates (Li et al., 2017). All statistical analyses of topological properties were corrected for multiple comparisons using the Bonferroni procedure with *p* < 0.01.

To further explore the indirect influence of anxiety on the relationship between network attributes and pain, mediating analysis was performed using the SPSS macro PROCESS (including bootstrapping) (Hayes, 2013). In the pain group, the identified topological attributes of brain functional networks were independent variables (X), STAI scores were intermediary variables (M), and VAS scores were dependent variables (Y), with age and gender as control variables. There are four paths: Path a (representing the relationship between X and M), Path b (representing the relationship between M and Y after controlling for X), Path c (representing the relationship between X and Y), and Path c’ (representing the relationship between X and Y after adjusting M), and the indirect impact is c-c’ (or a × b). Estimates of indirect effects are considered meaningful when zero is not included in the bootstrapped 95% confidence interval (CI) (5,000 iterations).

## Results

### Demographic and Clinical Characteristics

Four participants in the pain group were excluded for excessive head motion. Therefore, in the final analysis, the subjects included were 48 participants who received the experimental orthodontic pain intervention (21 males and 27 females, age 18-24 years with mean age 21.0 ± 1.1 years) and 49 controls who did not (22 males and 27 females, age 19-30 years with mean 21.0 ± 2.6 years). There were no significant differences in age (*t* = 0.05, *p* = 0.911), sex (*t* = 0.11, *p* = 0.961), and head motion (*t* = 1.29, *p* = 0.199) between the two groups. In the pain group, by paired *t*-test the pain severity (VAS score) was significantly higher after 24 h with the elastic separator (*t* = 2.45, *p* = 0.018). However, there was no difference in STAI scores before and after using the separator (*p* = 0.159) (Table 1). After 24 h with the elastic separator, partial correlation analysis showed a significant positive correlation between pain and anxiety (*r* = 0.62, *p* < 0.001) after adjusting for sex and age.

**TABLE 1:** Demographics and clinical data of participants with experimental orthodontic pain and control subjects.

Characteristic	Pain group (*n* = 48)		Controls (*n* = 49)	*p*
*Demographic factors*				
Sex (Male/Female)	21/27		22/27	0.911
Age (years)	21.02 ± 1.06		21.04 ± 2.62	0.961
*Clinical measurements*	Without separators	24 h with separators		
Visual analog scale (score)	14.6 ± 17.3	20.6 ± 17.4	−	0.018
State anxiety inventory (score)	28.1 ± 11.0	29.9 ± 11.4	−	0.159

### Alterations of Global Topological Organization

**FIGURE 1:**
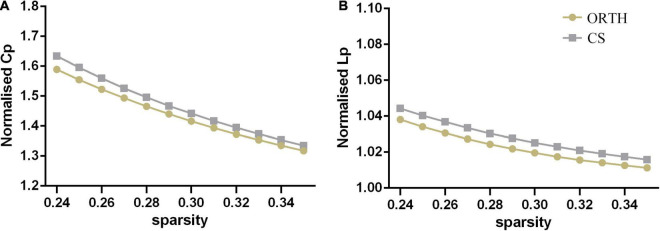
Both the orthodontic pain subjects (ORTH) and control subjects (CS) showed that **(A)** the normalized clustering coefficient (C_P_) was greater than 1 and **(B)** the normalized path length (L_P_) was approximately equal to 1, indicating that both groups showed typical small-world topological characteristics.

**FIGURE 2:**
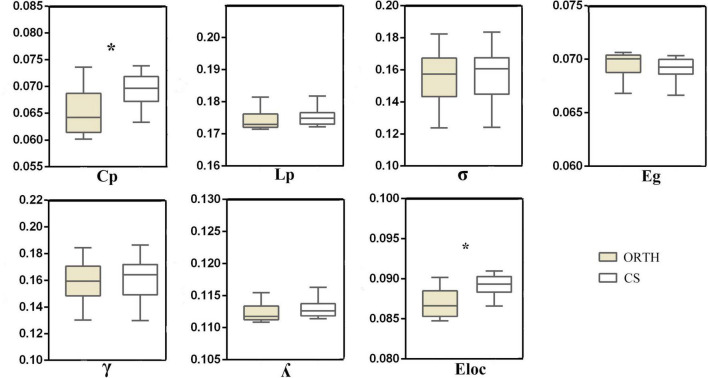
The topological properties of brain functional networks in orthodontic pain subjects (ORTH) and control subjects (CS), shown as the area under the curve across the sparsity range. Significant between-group differences were found in C_p_ (*t* = –5.43, *p* < 0.001) and E_loc_ (*t* = –6.62, *p* < 0.001). Abbreviations: C_p_, clustering coefficient; L_p_, characteristic path length; γ, normalized clustering coefficient; λ, normalized characteristic path length; σ, small-worldness; E_loc,_ local efficiency; E_g_, globe efficiency.

Both groups showed a small-world topology of brain resting-state functional organization (Figure 1). Compared with controls, the pain group showed significantly decreased clustering coefficient (*t* = −5.43, Bonferroni corrected *p* < 0.001) and local efficiency (*t* = −6.62, Bonferroni corrected *p* < 0.001) (Figure 2), with no significant differences in characteristic path length (*p* = 0.116), normalized clustering coefficient (*p* = 0.335), normalized characteristic path length (*p* = 0.066), small-worldness (*p* = 0.531), and global efficiency (*p* = 0.096).

### Alterations of Nodal Topological Organization

**FIGURE 3:**
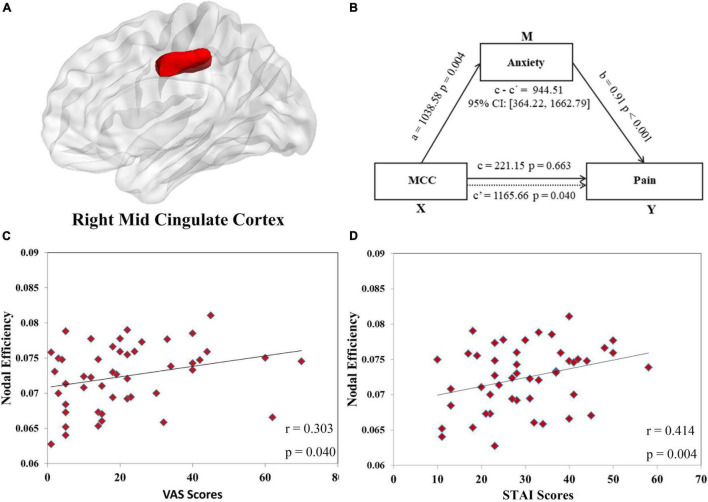
The positive correlations between the nodal efficiency of the right midcingulate cortex (MCC, shown in panel **(A)** and the visual analog scale (VAS) scores (*r* = 0.303, *p* = 0.040, shown in panel **(C)** and state anxiety inventory (SAI) scores (*r* = 0.414, *p* = 0.004, shown in panel **(D)** were found in orthodontic pain group. In mediation analysis, illustration **(B)** demonstrates that the node efficiency of MCC affects pain through anxiety in subjects with orthodontic pain, with sex and age controlled in the model.

A total of 23 brain regions were identified as showing significant differences between groups in at least one nodal metric (with Bonferroni corrections for multiple comparisons). Compared with controls, the nodal centrality of brain nodes ipsilateral to the right-sided pain stimulus was mainly increased, while that of contralateral brain nodes was decreased. Compared with controls, the *increased* nodal centralities (mainly ipsilateral) were in the superior frontal gyrus (SFG, right, lateral and middle areas), precentral gyrus (right, head and face region), parahippocampal gyrus (bilateral entorhinal cortex and right posterior cortex), postcentral gyrus (right), insula gyrus (right), basal ganglia (right caudal hippocampus and left ventral caudate) and thalamus (right occipital thalamus and bilateral caudal temporal thalamus). The *decreased* nodal centralities (mainly contralateral) were in the superior temporal gyrus (STG, left caudal area), middle temporal gyrus (left dorsolateral area), inferior temporal gyrus (left ventrolateral area), postcentral gyrus (left), cingulate gyrus [left and right (Figure 3A), middle area] and occipital cortex (left, middle ventral, inferior and lateral superior areas) (Table 2).

**TABLE 2:** Regions with altered nodal centralities in subjects with experimental orthodontic pain compared with control subjects.

Side	Brain region	Betweenness	Degree	Efficiency
Right	Superior frontal gyrus (lateral)	5.00*	3.40	3.95
Right	Superior frontal gyrus (medial)	3.04	4.30	4.53*
Right	Inferior frontal gyrus (ventral)	1.87	4.15	4.33*
Right	Precentral gyrus (head and face region)	5.45*	3.43	4.00
Right	Parahippocampal gyrus (entorhinal cortex)	3.53	4.49 *	4.13
Right	Postcentral gyrus	4.46*	1.43	1.85
Right	Insular gyrus	5.15*	3.90	4.52*
Right	Caudal hippocampus	1.65	5.43 *	4.62*
Right	Occipital thalamus	2.98	6.91*	6.57*
Left	Ventral caudate	2.02	4.48*	4.64*
Left	Caudal temporal thalamus	3.10	5.76*	5.22*
Left	Parahippocampal gyrus (entorhinal cortex)	2.59	5.09*	5.26*
Left	Parahippocampal gyrus (posterior area)	3.48	5.28*	4.82 *
Right	Middle cingulate gyrus	−3.91	−4.47*	−4.42*
Left	Superior temporal gyrus (caudal)	−4.50 *	−6.42*	−6.41*
Left	Middle temporal gyrus (dorsolateral)	−4.94 *	−7.53*	−7.34*
Left	Inferior temporal gyrus (ventrolateral)	−0.56	−5.03*	−4.72*
Left	Postcentral gyrus	−1.33	−4.58*	−4.19
Left	Middle cingulate gyrus	−4.91*	−4.82*	−5.08*
Left	Middle ventral occipital cortex	−0.96	−4.42*	−3.95
Left	Inferior occipital gyrus	−1.57	−4.47*	−4.22
Left	Lateral superior occipital gyrus	−2.98	−4.33*	−4.08
Right	Caudal temporal thalamus	4.59*	5.66*	5.46*

t values are shown, positive/negative t values suggesting the nodal centrality was larger/smaller than controls. *Regions were considered abnormal in participants with orthodontic pain than controls (p < 0.01, Bonferroni correction for multiple comparisons).

### Correlation and Mediation Analyses in the Pain Group

Exploring the relationships between topological properties (the two global characteristics in Figure 2 and 40 nodal metrics in Table 2 showing between-group differences) and clinical measurements (SAI and VAS scores at 24 h after application of the separator), we detected a positive correlation between VAS scores and nodal efficiency of right MCC (*r* = 0.303, uncorrected *p* = 0.040) (Figure 3C) and between STAI scores and nodal efficiency of right MCC (*r* = 0.414, uncorrected *p* = 0.004) (Figure 3D). However, these correlations did not survive Bonferroni correction.

In the first mediation analysis, we found a significant mediating effect of anxiety (M) on the relationship between nodal efficiency of right MCC (X) and pain (Y): indirect effect = 944.51; 95% CI = [364.22, 1662.79], *p* < 0.05 (Figure 3B) after adjusting for sex and age. To test the directionality of this relationship, we performed a second mediation analysis with anxiety as X, nodal efficiency of right MCC as M, and pain as Y. The results showed that nodal efficiency of right MCC (M) did not mediate the effect of the anxiety (X) on pain (Y): indirect effect = 0.04; 95% CI = [−0.14, 0.23], *p* > 0.05 after adjusting for sex and age. These findings implicate a single neuropsychological pathway: nodal efficiency of the right MCC influences pain via anxiety.

## Discussion

The whole-brain functional network topology exhibited a small-worldness both in subjects exposed to experimental orthodontic pain and controls. However, there were differences in the quantitative network parameters which can be summarized as decreased clustering coefficients and local efficiency in the pain group. Of the 23 nodes featuring 40 altered topological nodal properties in the pain group compared to controls, the nodal centrality of brain nodes ipsilateral to the pain stimulus was generally increased, while that of contralateral brain areas was decreased. The nodal efficiency of the right MCC was positively correlated with pain severity and anxiety, and mediation analysis finds that anxiety mediates the influence of MCC efficiency and pain intensity.

### Global Topological Organization

Both groups, as expected, showed a small-world organization, known to facilitate efficient inter-regional communication and to be resilient to network disruption (Watts and Strogatz, 1998). The present result is consistent with previous research in pain (Kaplan et al., 2019; De Pauw et al., 2020). Compared to controls, the clustering coefficient and local efficiency were significantly decreased in the orthodontic pain group. These two parameters represent the separation topology of networks, which refers to the ability of densely interconnected regions to perform specialized processing (Latora and Marchiori, 2001). This change implies a reduced fault tolerance of the network. Change of nodes or connections in the network will affect its overall processing capacity (Latora and Marchiori, 2001). Notably, there were no differences between groups in global efficiency and average path length, which are usually used to estimate the integration of brain networks (Latora and Marchiori, 2001), which refers to the efficiency of global information communication, or the ability to integrate distributed information (Latora and Marchiori, 2001). Taken together, our results indicate that brain functional networks in orthodontic pain become less efficient and less segregated, but that integration is not affected. In short, the whole brain network exhibited a randomized alteration in response to orthodontic pain.

### Nodal Topological Organization

At the regional level, we found nodal centrality increased mainly on the right side of the brain hemispheres, including SFG, precentral gyrus, parahippocampal gyrus, postcentral gyrus, insula, basal ganglia and thalamus. The insula plays an important role in “internal receptive” pain perception (Craig, 2003, 2004), and this finding implies an increase in information transmission efficiency of the insula. This is in line with previous reports of increased insular activity in pain (Jantsch et al., 2005), and suggests that the orthodontic pain stimulus may activate insula to produce and transmit pain sensation. Since the nociceptive input of insula comes from thalamus (Craig et al., 1994), and the ipsilateral activity of thalamus stimulated by pain was also increased, the high activity of ipsilateral insular lobe may reflect the upward conduction pathway of ipsilateral injury. In addition, there is evidence that pain information can be sent by the insula to SFG (Bantick et al., 2002). The SFG shows an inverse coding, i.e., stronger responses to weaker pain stimuli (Jantsch et al., 2005), and there is a negative correlation between pain intensity and activation of SFG (Bantick et al., 2002). Interestingly, we found that the nodal centrality of the right SFG was increased, while that of the left SFG was decreased; perhaps the facial stimulation information is received in the bilateral gyrus (Cusick et al., 1986) but the response differs between the stimulation side and the contralateral side.

We also found increased nodal centrality in the right thalamus. To some degree, this agrees with previous results of pain-related thalamus activation (Farrell et al., 2005). The strong ipsilateral thalamic activity associated with pain may reflect the thalamocortical input from the brainstem on receiving the ascending nociceptive input (Millan, 1999). The thalamus-somatosensory pathway plays an important role in pain stimuli (Walton et al., 2010; Youssef et al., 2014). Since the postcentral gyrus is the main part of the somatosensory cortex, the differential activation of bilateral posterior central gyrus may be related to the different projections of the fiber bundles to the thalamus (Millan, 1999; Ohara and Lenz, 2003). The precentral cortex receives inputs from the thalamus and somatosensory cortex. In line with previous work (Jantsch et al., 2005) and the ipsilateral pain transmission findings described above, we found increased nodal properties in the right precentral cortex. This activation reflects the focusing of attention on the pain inputs (Youssef et al., 2014) and indicates that managing orthodontic pain may be a more demanding neural task (Jantsch et al., 2005). We also found increased nodal centrality in the parahippocampal gyrus. According to previous work, pain stimuli not only transmit to the sensory cortex but also include memory-related regions (Long et al., 2016). In line with previous results (Gondo et al., 2012), the present finding may reflect how the pain experienced will be remembered and compared to previous pain (Bergius et al., 2002).

Similar to two studies of clinical pain (rheumatoid arthritis and migraine) (Liu et al., 2012; Basu et al., 2019), decreased nodal centrality was also found in the temporal gyrus, occipital cortex, and MCC. Interestingly, most of the findings were in the left hemisphere contralateral to the pain stimulation, reflecting the inconsistent functional nodal centralities of the bilateral cortex. The orofacial pain study reported decreased nodal centrality of temporal gyrus in patients (Kaplan et al., 2019) and the gray matter volume disruption of temporal gyrus was negatively correlated with pain intensity (Wang et al., 2017). The temporal gyrus may play a role in the memory processes related to the emotional components of pain, which may be related to the memory impairment of patients with orofacial pain (Vincent and Hadjikhani, 2007; Gondo et al., 2012). The occipital cortex is the main region of the visual network (Kaplan et al., 2019). Previous pain studies found that the nodal centrality of visual network related regions was decreased (Kaplan et al., 2019) and in unpleasant visual stimulation the pain-related brain activity increased (Harte et al., 2016). Thus the decreased nodal centrality of the occipital cortex may reduce the sensitivity of pain patients to vision-related unpleasant feelings (Kaplan et al., 2019). However, the role of temporal and occipital cortex in imaging studies of pain needs further investigation.

In these regions with reduced nodal centralities, the right MCC was positively correlated with VAS scores. The MCC is involved in pain intensity coding (Mohr et al., 2005), and the contralateral (right) MCC has the characteristics of inverse intensity coding (Jantsch et al., 2005) (the more severe the pain, the less activity). This is consistent with our findings: the pain was increased at 24 h, but the functional properties of MCC were decreased. The decreased node centrality of MCC may also be a maladaptation to pain stimuli. The MCC plays an important role in sensory integration during pain processing (Bornhövd et al., 2002), comparing the predicted sensory results of pain stimulation with the actual sensory feedback (Blakemore et al., 1998). The decreased functional properties of MCC may reflect the mismatch between pain prediction and sensory feedback (Mohr et al., 2005).

In conclusion, our study found that MCC is involved in the pain intensity coding and the sensory integration of pain. However, the correlation is only moderate and did not survive corrections for multiple comparisons. It will be important to explore the neural mechanism of MCC in pain in longitudinal studies.

### Implications of Mediation Analysis

We found that anxiety plays a mediating role in the relationship between the nodal efficiency of right MCC and pain. Patients suffer a high level of anxiety and pain during orthodontic treatment (Sari et al., 2005), and pain can be increased by anxiety (Reicherts et al., 2017). We also found the pain was positively correlated with anxiety. Moreover, hierarchical regression analysis showed that anxiety had an incremental predictive ability for pain (Δ*R^2^* = 36.5%, β = 0.61, *p* < 0.001) even after controlling for age and sex. At the neural level, a significant positive correlation was found between nodal efficiency of right MCC and anxiety. The MCC is involved in negative affect (Wang et al., 2017). Anxiety under pain stimulation is positively related to the activity of MCC (Reicherts et al., 2017; Wang et al., 2017). However, the decreased functional properties of MCC in the pain group may be a functional compensatory response: the greater the anxiety, the stronger the compensation represented by a lower nodal efficiency of right MCC (Yang et al., 2015). As mentioned, MCC participates in the feedforward component of sensory integration (Blakemore et al., 1998), and so the decreased nodal efficiency of right MCC may reflect the imbalance between the actual anxiety and the individual’s expected anxiety, and individuals may adapt to anxiety and pain by reducing the function of MCC. Taking this evidence together, anxiety caused by the alteration of functional properties in MCC may be an important neuropsychological mechanism for aggravating or causing orthodontic pain.

### Limitations

**Although the sample size is larger than most studies of orthodontic pain, it may still have limited the power of the study.** This study was cross-sectional, and the mediation analysis is of course statistical, and does not guarantee causality. In the future, more complex methods (such as longitudinal designs) will be needed to determine the causal direction of the relationship between these variables. **Besides, present study found altered node attributes related to the location of the pain stimulus, however, only subjects with right pain stimulation are included. Further study with left pain stimulation is needed to prove the result.** More importantly, a future study needs to consider pain-related emotional changes and their relationship with brain functional alterations.

## Conclusion

The present study identified global and local changes in brain functional network topology in experimental orthodontic pain. The participants with pain stimulation showed a preserved small-world functional network organization, but with altered node attributes related to the location of the pain stimulus. Ipsilateral (right) pain-related brain region activity was activated, and most of these areas have inverse coding characteristics, activation resulting in reduced pain perception. The activity of the contralateral (left) brain area was decreased. Furthermore, anxiety mediates the relationship between MCC and pain, suggesting a potential neuropsychological mechanism. Our results may assist the targeted selection of interventions for orthodontic pain (such as behavioral (Lichtenstein et al., 2018) or brain interventions (Boecker et al., 2008; Esch and Stefano, 2010; Tuulari et al., 2018; Fontes et al., 2020), to help reduce pain and improve cooperation during orthodontic treatment (Li et al., 2020).

## Data Availability Statement

The raw data supporting the conclusions of this article will be made available by the authors, without undue reservation.

## Ethics Statement

The studies involving human participants were reviewed and approved by West China Hospital Ethics Committee of Sichuan University. The patients/participants provided their written informed consent to participate in this study.

## Author Contributions

FZ, FL, WL, HY, ZJ, and QG conceived and designed the experiments. FZ, FL, HY, and YJ performed the experiments and analyzed the data. FZ, FL, and GK wrote and critically reviewed the first draft of the manuscript. All authors contributed to the writing, critical review and approval of the final manuscript.

## Conflict of Interest

The authors declare that the research was conducted in the absence of any commercial or financial relationships that could be construed as a potential conflict of interest.

## Publisher’s Note

All claims expressed in this article are solely those of the authors and do not necessarily represent those of their affiliated organizations, or those of the publisher, the editors and the reviewers. Any product that may be evaluated in this article, or claim that may be made by its manufacturer, is not guaranteed or endorsed by the publisher.

## Footnotes

1
http://www.fil.ion.ucl.ac.uk/spm

2
http://rfmri.org/DPARSF

3
http://www.nitrc.org/projects/gretna/
